# Editorial: Recent advances in pediatric red blood cells disorders

**DOI:** 10.3389/fped.2024.1403651

**Published:** 2024-04-03

**Authors:** Gabriele Canciani, Giuseppe Palumbo, John Brewin, Francesca Rossi, Giulia Ceglie

**Affiliations:** ^1^ Hematology/Oncology, Cell and Gene Therapy, IRCCS Bambino Gesù Children’s Hospital, Rome, Italy; ^2^ Residency School of Pediatrics, University of Rome Tor Vergata, Rome, Italy; ^3^ Department of Systems Medicine, University of Rome Tor Vergata, Rome, Italy; ^4^ Department of Haematological Medicine, King’s College Hospital, London, United Kingdom; ^5^ Department of Woman, Child and of General and Specialist Surgery, University of Campania Luigi Vanvitelli, Naples, Italy

**Keywords:** pediatric hematology, red blood cells disorders, hemoglobinopathies, spherocytosis, gene-editing

**Editorial on the Research Topic** Recent advances in pediatric red blood cells disorders

Red blood cell (RBC) disorders are among the most prevalent human diseases and encompass a heterogenous group of conditions, including hemoglobinopathies and erythrocytes' enzymatic or structural defects. The majority of RBC disorders are genetically inherited and typically present in early-childhood, thus reflecting a pivotal role of the pediatricians for promptly recognizing and differentiating these conditions. In this regard, our research topic offers interesting contributions, with a particular focus on diagnostic methodologies and management strategies for RBC disorders.

Hemoglobinopathies represent the most common monogenic diseases (1, 2). They can be categorized in two principal groups: structural hemoglobinopathies, characterized by abnormal hemoglobin's functionality, and thalassemias, resulting from inadequate synthesis of globin chains. These genetic disorders are typically inherited in an autosomal recessive fashion and, notably, almost 7% of the world population carries at least one pathogenetic globin genes' allele (3). The mutational landscape of these conditions is wide and, to date, more than 1.000 pathogenetic variants in globing genes have been described (1). Due to globalization and migratory flows, the epidemiology of hemoglobinopathies is evolving with new emerging combinations of variants, thus intricating the multifaceted presentation of these diseases, ranging from asymptomatic to transfusion dependent conditions (4, 5). In light of this complex framework, Marchesani et al. report an interesting description of blood count morphological parameters as simple predictive indicators in order to identify children most likely to have an underlying genetic disorder, thus providing important efficiencies to the role of confirmatory genetic tests. In details, patients with anemia carrying mutated alleles in the globin genes displayed significant variations in counts and mean volumes of either RBC and reticulocytes as compared to patients with negative genetic screening. These blood count parameters were able to predict the presence of a pathogenetic variant, even in case of negative study of hemoglobin variants by means of high-performance liquid chromatography, thus unveiling the possibility to use such widely available markers for initial screening. Interestingly, the group of Turudic et al. reported two composite indices aimed at differentiating non-transfusion dependent β-thalassemia and iron deficiency anemia in children. The latter, being one of the most form of anemia in children, must always be taken into account when considering differential diagnoses with hemoglobinopathies. These two studies highlighted the importance of laboratory tests and derived indices in case of suspected hemoglobinopathies presenting mild phenotypes; however, genetic analysis remain the mainstay for confirmatory diagnosis. In this regard, Zhan et al. provide a concise and insightful framework on the application of third generation sequencing for the diagnosis of β-thalassemia. This innovative technology, which has not entered routine clinical practice yet, offers advantages in terms of sensibility and diagnostic accuracy as compared to currently available methodologies. In future, implementations of the genetic screening will be crucial in order to detect genetic variants and possibly improve our capacity to correlate patients' phenotypes and genotypes (6). Moreover, advances in genetic technologies are not only useful to establish an accurate diagnosis, but also paved the way for potentially curative interventions for hemoglobinopathies (7, 8). Recently, the Food and Drug Administration (FDA) approval of the first gene-editing based therapies for Sickle Cell Disease (SCD) marked a significant milestone in this field (9). Specifically, this revolutionary approach is based on the clustered regularly interspaced short palindromic repeats (CRISPR)/CRISPR-associated protein 9 (Cas9) mediated disruption of B-cell lymphoma/leukemia 11A (BCL11A) gene, which is a transcriptional repressor of the gamma globin gene, responsible for production of Fetal Hemoglobin (HbF). The resulting elevated levels of this alternative hemoglobin induce an impressive amelioration of clinical outcomes in both SCD and β-thalassemia (10). However, this nuclease-based approach might entail unpredictable genotoxic risks due to the double-strand break on the DNA. In this regard, Ceglie et al. report an extensive review discussing the innovative gene-editing therapeutic strategies for SCD, narrowing their attention to the innovative nuclease-free genome editing technologies which might further optimize the safety profile of these promising treatments.

Besides hemoglobinopathies, RBC disorders might arise from structural abnormalities of erythrocytes, such as Hemolytic Spherocytosis (HS). This chronic hemolytic anemia arises from impaired RBC stability and deformability due to alterations in proteins composing the delicate connections between cytoskeletal and membrane. Although rare occurring in around 1 in every 2,000 individuals, HS is the most prevalent hemolytic anemia in Northern European and North American countries (11). Interestingly, Boaro et al. highlighted the importance of international networks, such as the EuroBloodNet network, in order to standardize and improve the diagnostic capacity of local centers. Moreover, they report a high incidence of HS-related hepatobiliary disorders in young age, unveiling the importance of optimizing the pediatric management of HS by anticipating the screening of potential complications.

Lastly, our editorial topic presents two peculiar pediatric case reports. Giannini et al. reported an interesting erythrocytosis case ultimately due to hypermagnesemia with dystonia 1 (HMNDYT1). This extremely rare disease is caused by mutations in the *SLC30A10* gene, encoding for a manganese transporter, thus inducing hypermagnesemia, erythrocytosis, dystonia and parkinsonism. Interestingly, the child exhibited isolated polycythemia without any evident neurological manifestation. Hence, a chelation therapy was immediately started with the aim of potentially mitigating the neurodegeneration. The group of Turudic et al. describe the case of a patient with autoimmune hemolytic anemia (AIHA), probably triggered by recent viral infections, presenting both cold and warm autoantibodies inducing a potent activation of both classical and alternative complement pathways. The child required an intense immunosuppressive therapy and the genetic analysis revealed an intronic mutation in the gene of C3, previously associated with alternative complement pathways disorders that possibly explains the devastating immunological reaction.

Overall, this topic offers an insightful framework of the multifaced clinical aspects of RBC disorders in pediatric age. In future, international networks will be crucial in order to improve our knowledge about genetic background, phenotypical correlations and clinical management of this heterogeneous group of diseases. Moreover, the landscape of gene-editing is undergoing a remarkable expansion, thus potentially offering revolutionary curative treatments to patients afflicted by genetic diseases, including pediatric RBC disorders.
